# Physical Rehabilitation and Post-Stroke Pneumonia: A Retrospective Observational Study Using the Japanese Diagnosis Procedure Combination Database

**DOI:** 10.3390/neurolint15040094

**Published:** 2023-12-04

**Authors:** Takehiro Nishimura, Ryutaro Matsugaki, Shinya Matsuda

**Affiliations:** 1 Department of Preventive Medicine and Community Health, University of Occupational and Environmental Health, Kitakyushu 807-8555, Japan; nsmr@med.uoeh-u.ac.jp (T.N.); smatsuda@med.uoeh-u.ac.jp (S.M.); 2 Department of Work Systems and Health, Institute of Industrial Ecological Sciences, University of Occupational and Environmental Health, Kitakyushu 807-8555, Japan

**Keywords:** ischemic stroke, Japan, physical rehabilitation, pneumonia

## Abstract

In this study, the relationship between the duration of physical rehabilitation and occurrence of pneumonia after ischemic stroke was examined. We included 426,508 patients aged ≥75 years with acute ischemic stroke. A multilevel logistic regression analysis nested at the hospital level was conducted to examine the association between the duration of physical rehabilitation and occurrence of pneumonia. The duration of physical rehabilitation refers to the hours of physical rehabilitation performed daily until the 7th day of hospitalization. In the multivariable analysis, the intensity of rehabilitation for durations of 20–39 min/day (adjusted odds ratio [aOR]: 0.78, 95% Confidence Interval [CI]: 0.75–0.81, *p* < 0.001), 40–59 min/day (aOR: 0.68, 95% CI: 0.66–0.71, *p* < 0.001), 60–79 min/day (aOR:0.56, 95% CI: 0.53–0.58, *p* < 0.001), and ≥80 min/day (aOR: 0.46, 95% CI: 0.44–0.48, *p* < 0.001) were significantly associated with a reduced incidence of pneumonia. In addition, the trend identified for duration of rehabilitation was significant (*p* < 0.001). The results of this study suggest the usefulness of high-duration physical rehabilitation for preventing pneumonia in older patients with ischemic stroke.

## 1. Introduction

Pneumonia is one of the most common stroke complications. Its incidence after stroke is estimated to be 3.2–14.3% [1,2,3], with most cases occurring within 7 days of hospitalization [3]. Older age [4], male sex [4], stroke severity [4,5], comorbidity (including chronic obstructive pulmonary disease, angina, or coronary artery disease) [4], dysphasia [4,5,6,7,8], dependency before admission [4], low mobility on admission [9], mechanical ventilation [5], and nasogastric tube [5,9] use are known to increase the risk of pneumonia and respiratory complications after stroke. Pneumonia prevention is of upmost importance because pneumonia is associated with undesirable outcomes, such as increased length of hospitalization [4,10,11], poor functional outcomes [3,4,10], increased costs of hospitalization [12], reduced frequency of routine home discharge [2], serious complications (such as sepsis), and mortality [2,3,4,10,11].

Physical rehabilitation is one of the most common treatments for stroke. Rehabilitation programs should be individualized according to the pathophysiology of the stroke within 24–48 h of admission to prevent complications and enhance functional recovery [13,14,15]. Poststroke physical rehabilitation generally includes swallowing training, early mobilization, and exercise interventions to improve disability by physical, occupational, and speech therapists, which are thought to contribute to the prevention of pneumonia [16,17,18].

Recently, high-duration physical rehabilitation has been shown to contribute to improved outcomes after ischemic stroke. An observational study using Japanese claims-data reported that higher-duration physical rehabilitation contributed to improved activities of daily living [19]. We hypothesized that this dose-response relationship would also apply to pneumonia as an outcome, although the relationship between rehabilitation intensity and the occurrence of pneumonia after ischemic stroke remains unclear. In this study, we examined the relationship between the duration of physical rehabilitation and occurrence of pneumonia after ischemic stroke using the Japanese Diagnosis Procedure Combination (DPC) database. We hypothesized that physical rehabilitation duration is associated with a lower incidence of pneumonia.

## 2. Materials and Methods

### 2.1. Study Design and Data Sources

This observational study utilized data from the Japanese DPC database. DPC is the main medical service reimbursement system for acute inpatient care in Japan, launched by the Ministry of Health, Labour and Welfare of Japan in 2002 [20]. The DPC database consists of information relevant to that system and contains the following information concerning hospitalized patients: (1) patient demographics such as sex, age, and admission-discharge pathways; (2) information pertaining to the diagnosis; and (3) details of the medical procedures performed during hospitalization. Importantly, the DPC data used were collated by the DPC research group and represents approximately 90% of the tertiary emergency hospitals in Japan [21]. In this study, we used case data of patients discharged between April 2014 and March 2020. This study was approved by the Institutional Review Board of the University of Occupational and Environmental Health, Japan (approval number: R4-045).

### 2.2. Study Participants

**Figure 1:**
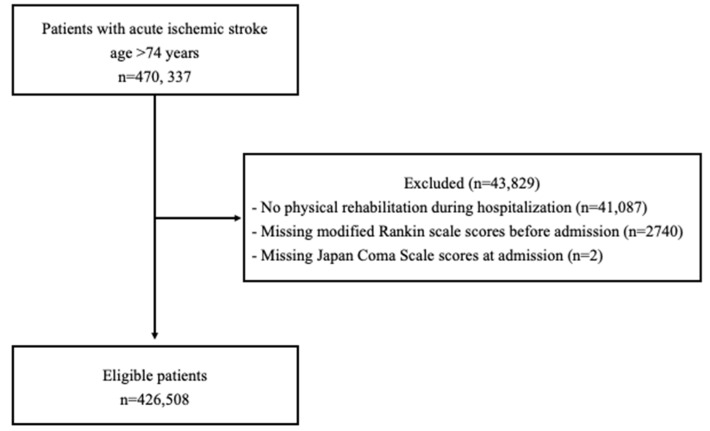
Flowchart of study inclusion.

We included individuals aged 75 years or older with acute ischemic stroke (International Classification of Diseases, Tenth Revision [ICD-10 code]: I63) who were hospitalized within three days of stroke onset (n = 470,337). Patients who did not undergo rehabilitation during hospitalization and those with an unknown modified Rankin Scale score prior to admission or Japan Coma Scale score at admission were excluded. Of the 470,337 patients identified, 426,508 were included in the analysis (Figure 1).

### 2.3. Assessment of Physical Rehabilitation Duration

Physical rehabilitation was provided by medical doctors and physical, occupational, and speech therapists under Japan’s national healthcare insurance system. Each session lasted 20 min, and patients could undergo up to 180 min of rehabilitation per day. In previous studies, the duration of physical rehabilitation was determined by dividing the total number of hours of rehabilitation provided during a hospital stay by the number of days spent in the hospital [19,22]. Considering that pneumonia after stroke in this study often occurred within seven days [3], the duration of physical rehabilitation was calculated up to the 7th day of hospitalization. We divided the duration of physical rehabilitation into five groups: <20, 20–39, 40–59, 60–79, ≥80 min/d.

### 2.4. Assessment of Pneumonia

Pneumonia, including aspiration pneumonia, was identified using the ICD-10 codes J12-J18 and J69 for complications during hospitalization in acute care hospital [10,23].

### 2.5. Assessment of Covariates

The following variables were examined in this study: age, sex, subtype of ischemic stroke (arterial thrombosis, arterial embolism, and others) [23], Charlson comorbidity index (CCI) score [24,25], Japan Coma Scale (JCS) score at admission [26,27], modified Rankin Scale (mRS) score before admission [28], type of acute care implemented (intravenous thrombolysis, and endovascular thrombectomy), fiscal year (2014, 2015, 2016, 2017, 2018, and 2019), and hospital case volume.

The CCI was introduced by Charlson et al. in 1987 as a method to quantify comorbid conditions [24]. It evaluates 17 comorbid conditions, such as myocardial infarction and congestive heart failure, based on ICD-10 codes [25]. The index assigns weights to each condition, with the aggregate of these scores constituting the CCI score. In this study, the CCI scores were categorized into four groups: 0, 1, 2, and ≥3.

The JCS is a metric predominantly utilized in Japan for assessing the level of consciousness in patients [26,27]. It categorizes consciousness impairment into nine levels based on severity, with a score of 0 indicating no impairment. For patients who are awake without any stimuli, a single-digit score (1, 2, 3) is assigned. If the patient can be aroused but reverts to their previous state after stimulation ceases, a two-digit score (10, 20, 30) is used. For patients who cannot be aroused with any forceful mechanical stimuli, a three-digit score (100, 200, 300) is designated. In this study, scores were classified into four categories based on this system: 0 for alert consciousness; 1–3 for wakefulness without stimuli; 10–30 for arousal by some stimuli; and 100–300 for coma. The JCS score can be converted to the internationally used Glasgow Coma Scale (GCS) score. For instance, a JCS score of 3 corresponds to a GCS score of 13, a JCS score of 30 corresponds to a GCS score of 9, and a JCS score of 300 corresponds to a GCS score of 3 [29].

The mRS is a scale used to assess the degree of disability and independence in patients who have suffered a stroke [28]. The mRS is a six-tiered metric ranging from 0 to 5, where 0 indicates no symptoms; 1 signifies no significant disability; 2 represents slight disability; 3 denotes moderate disability; 4 corresponds to moderately severe disability; and 5 reflects severe disability.

### 2.6. Statistical Analysis

Continuous variables, such as age, were presented as the median and interquartile range. categorical variables were expressed as numbers and percentages.

In the main analysis, a multilevel logistic regression analysis (nested at the hospital level) was conducted, with pneumonia as the dependent variable and the duration of physical rehabilitation as the independent variable. To adjust for potential confounders, we used age, sex, ischemic stroke subtype, CCI score, JCS score at admission, mRS score before admission, acute care type, fiscal year, and hospital case volume as covariates.

As a supplementary analysis, multilevel logistic regression analysis (nested at the hospital level) was conducted by classifying pneumonia into aspiration pneumonia (J69) and other pneumonia (J12-18) [30], with these as the dependent variables and the duration of physical rehabilitation as an independent variable. The covariates were the same as those used in the main analysis.

All statistical analyses were performed using Stata software (Stata Statistical Software: Release 18; StataCorp LLC, College Station, TX, USA). A *p*-value of <0.05 were considered statistically significant.

### 2.7. Sensitivity Analysis

We conducted the following sensitivity analyses: First, while our primary analysis adopted the duration of physical rehabilitation within the first seven days of hospitalization as the exposure, we performed sensitivity analyses to validate the robustness of our findings. For this purpose, we redefined exposure as the duration of physical rehabilitation up to three and five days post-admission. Second, we hypothesized that the association between the duration of physical rehabilitation and the onset of pneumonia would vary depending on the severity of the patient’s consciousness impairment. To examine potential effect modifications, we conducted stratified sensitivity analyses based on the severity of patients’ consciousness impairment. Finally, in patients requiring mechanical ventilation shortly after admission, implementing high-duration physical rehabilitation is challenging. Mechanical ventilation is a known risk factor for pneumonia onset. Given that this could introduce bias in our results, we conducted a sensitivity analysis excluding 3227 patients who required mechanical ventilation within the first 2 days of admission, focusing on the remaining 423,281 patients.

## 3. Results

Table 1 shows the characteristics of the participants according to the duration of physical rehabilitation. The proportion of patients with a Japan Coma Scale score ranging from 100 to 300 decreased as the duration of physical rehabilitation increased: <20 min/d (11.1%), 20–39 min/d (5.9%), 40–59 min/d (3.6%), 60–79 min/d (2.5%), and ≥80 min/d (1.9%). Similarly, the proportion of patients with a mRS score of 5 before admission also declined with increasing duration of physical rehabilitation: <20 min/d (9.3%), 20–39 min/d (5.9%), 40–59 min/d (5.0%), 60–79 min/d (4.6%), and ≥80 min/d (4.4%).

Table 2 shows the incidence of pneumonia according to the duration of physical rehabilitation. The occurrence of pneumonia decreased with increasing duration of rehabilitation: <20 min/d (13.4%), 20–39 min/d (10.1%), 40–59 min/d (8.9%), 60–79 min/d (7.4%), and ≥80 min/d (6.4%). This trend was consistent when pneumonia was divided into non-aspiration and aspiration pneumonia.

The adjusted odds ratios (aORs) for pneumonia associated with the physical rehabilitation duration are shown in Table 3. In the multivariable analysis, physical rehabilitation durations of 20–39 min/day (aOR: 0.78, 95% CI: 0.75–0.81, *p* < 0.001), 40–59 min/day (aOR: 0.68, 95% CI: 0.66–0.71, *p* < 0.001), 60–79 min/day (aOR: 0.56, 95% CI: 0.53–0.58, *p* < 0.001), and ≥80 min/day (aOR: 0.46, 95% CI: 0.44–0.48, *p* < 0.001) were significantly associated with a reduced incidence of pneumonia. Furthermore, the analysis indicated a significant trend for the duration of physical rehabilitation (*p*-value for trend <0.001).

Table 4 presents the supplemental analysis results when patients were categorized into the non-aspiration and aspiration pneumonia groups. Consistent with the primary analysis, a higher duration of physical rehabilitation was associated with a decreased incidence of both non-aspiration and aspiration pneumonia (*p* < 0.001).

We performed three sensitivity analyses, and in each analysis, the association between the duration of physical rehabilitation and the occurrence of pneumonia was similar to that in the main analysis (Tables S1–S3).

## 4. Discussion

We examined the relationship between physical rehabilitation duration and the incidence of pneumonia in older stroke patients using Japanese DPC data. The results revealed that a higher physical rehabilitation duration was associated with a lower incidence of pneumonia, and a dose-response relationship was also confirmed. The results of this study suggest the usefulness of high-duration physical rehabilitation for pneumonia prevention in older patients with stroke.

This study has two main strengths. First, it uses a database that covers approximately 90% of all tertiary emergency hospitals in Japan [21,31]. This may contribute to the generalizability of the results of this study. Second, the study showed a dose-response relationship between the duration of physical rehabilitation and the prevention of pneumonia. Although several findings have suggested that physical rehabilitation is important in preventing the occurrence of pneumonia [32], the novelty of this study lies in demonstrating the significance of the intensity of physical rehabilitation.

Our results suggest that patients with ischemic stroke who receive high-duration physical rehabilitation have a lower incidence of pneumonia. Another observational study in Japan suggested that high-duration physical rehabilitation was associated with independence in activities of daily living [19]. The results of this study suggest that high-duration physical rehabilitation for patients with ischemic stroke not only improves activities of daily living, but may also contribute to the prevention of pneumonia, thus emphasizing the clinical significance of intensive physical rehabilitation. From another perspective, our results complement those of previous studies in that high-duration physical rehabilitation may contribute to improving patients’ activities of daily living by reducing the risk of pneumonia.

The mechanism by which rehabilitation suppresses pneumonia has not been elucidated. There may be two possible mechanisms for this phenomenon. One is the prevention of aspiration through early assessment and training of swallowing by a speech-language pathologist. A previous meta-analysis reported a prevalence of 42% for post-stroke dysphagia (PSD) and a 4.35-fold increased risk of pneumonia in the group with PSD compared to the group without PSD [7]. This suggests that the prevention and treatment of dysphagia are important for preventing the development of post-stroke pneumonia. Early dysphagia screening and swallowing therapy have been reported to be useful in preventing pneumonia [18,33]. Although debate continues regarding which method is the best for swallowing screening, recent systematic reviews suggest the usefulness of the Bedside Aspiration test, Gugging Swallowing Screen and Toronto Bedside Swallowing Screening Test [34]. Another possibility is that early mobilization and exercise therapy for disabilities by occupational and physical therapists may have improved low mobility, which is a risk factor for respiratory complications, and that early mobilization may have improved the impaired consciousness and dysphagia [17]. In addition, various interventions, including mobilization, positioning, and breathing exercises, have been recommended to eliminate retained airway secretions in critically ill patients [35]. These interventions may promote the removal of airway secretions, reduce the occurrence of atelectasis, and decrease the risk of pneumonia.

This study has a few limitations. First, in studies using DPC data, the type of physical rehabilitation and timing of pneumonia onset remain unknown. Therefore, although the study demonstrated the benefit of physical rehabilitation, the specific type of physical rehabilitation that is beneficial and the combination of physical rehabilitation that is appropriate, remains unknown. Previous studies have demonstrated the effectiveness of multidisciplinary team interventions [17]. Therefore, a balanced intervention consisting of speech, occupational, and physical therapies is desirable. Second, there are unmeasured confounding factors. A major limitation of this study is the lack of adjustment for stroke severity at onset, as assessed by the NIHSS score, which is associated with functional disability, mortality, and occurrence of pneumonia after ischemic stroke [2,36,37,38]. Patients with a higher severity of ischemic stroke may receive a shorter duration of physical rehabilitation, and concurrently, exhibit an increased frequency of pneumonia occurrence. This scenario suggests that if the severity of stroke, a potential confounding factor, is not adequately controlled for, it could lead to an overestimation of the protective effect of prolonged physical rehabilitation on the risk of pneumonia. However, we adjusted for variables that are surrogates for stroke severity, such as conscious state at admission and stroke type. In addition to stroke severity, detailed classification of ischemic stroke and the presence of dysphasia are also unmeasured confounding factors. Third, it is known that DPC data have high specificity but low sensitivity for diagnoses [39]. The possibility of misclassification of pneumonia, which is the outcome, cannot be denied. If misclassification of the outcome is occurring, the association between the duration of physical rehabilitation and pneumonia might be underestimated. Finally, the possibility of reverse causality cannot be ruled out, because the timing of pneumonia onset could not be specified in this study. However, we conducted a sensitivity analysis excluding patients who were ventilated soon after hospitalization and a sensitivity analysis using the rehabilitation duration within a very short period of time after hospitalization (within 3 days) as the exposure and confirmed that the relationship between rehabilitation intensity and the occurrence of pneumonia remained the same even when the situations in which reverse causality occurred were controlled as much as possible. The relationship between rehabilitation duration and the occurrence of pneumonia remained unchanged even when situations in which reverse causality occurred were controlled as much as possible.

## 5. Conclusions

Increasing the duration of rehabilitation in older patients with stroke may reduce the risk of developing pneumonia. This finding underscores the need for active rehabilitation in the acute phase of ischemic stroke. Healthcare providers should intensify their rehabilitation efforts to prevent pneumonia after ischemic stroke.

## Figures and Tables

**Table 1:** Participant Characteristics.

		Duration of Physical Rehabilitation				
	Total (n = 426,508)	<20 min/Day (n = 43,505)	20–39 min/Day (n = 125,126)	40–59 min/Day (n = 123,718)	60–79 min/Day (n = 73,172)	≥80 min/Day (n = 60,987)
Age, median (IQR)	83.0 (79.0, 88.0)	84.0 (79.0, 88.0)	83.0 (79.0, 88.0)	83.0 (79.0, 88.0)	83.0 (79.0, 88.0)	83.0 (79.0, 87.0)
Sex						
men	206,564 (48.4%)	20,208 (46.4%)	60,618 (48.4%)	60,210 (48.7%)	35,860 (49.0%)	29,668 (48.6%)
women	219,944 (51.6%)	23,297 (53.6%)	64,508 (51.6%)	63,508 (51.3%)	37,312 (51.0%)	31,319 (51.4%)
Subtype of ischemic stroke						
Arterial thrombosis	160,613 (37.7%)	14,021 (32.2%)	45,424 (36.3%)	47,311 (38.2%)	29,146 (39.8%)	24,711 (40.5%)
Arterial embolism	134,911 (31.6%)	15,827 (36.4%)	41,543 (33.2%)	38,997 (31.5%)	21,323 (29.1%)	17,221 (28.2%)
Others	130,984 (30.7%)	13,657 (31.4%)	38,159 (30.5%)	37,410 (30.2%)	22,703 (31.0%)	19,055 (31.2%)
Charlson comorbidity index						
0	142,996 (33.5%)	14,669 (33.7%)	42,495 (34.0%)	41,323 (33.4%)	24,559 (33.6%)	19,950 (32.7%)
1	134,435 (31.5%)	13,761 (31.6%)	39,641 (31.7%)	39,556 (32.0%)	23,128 (31.6%)	18,349 (30.1%)
2	85,057 (19.9%)	8519 (19.6%)	24,682 (19.7%)	24,616 (19.9%)	14,558 (19.9%)	12,682 (20.8%)
3≤	64,020 (15.0%)	6556 (15.1%)	18,308 (14.6%)	18,223 (14.7%)	10,927 (14.9%)	10,006 (16.4%)
Japan Coma Scale score at admission						
0	157,335 (36.9%)	14,341 (33.0%)	46,992 (37.6%)	45,397 (36.7%)	27,662 (37.8%)	22,943 (37.6%)
1–3	200,509 (47.0%)	17,503 (40.2%)	54,944 (43.9%)	59,339 (48.0%)	36,803 (50.3%)	31,920 (52.3%)
10–30	48,999 (11.5%)	6816 (15.7%)	15,832 (12.7%)	14,471 (11.7%)	6894 (9.4%)	4986 (8.2%)
100–300	19,665 (4.6%)	4845 (11.1%)	7358 (5.9%)	4511 (3.6%)	1813 (2.5%)	1138 (1.9%)
Modified Rankin Scale score before admission						
0	163,342 (38.3%)	15,442 (35.5%)	47,664 (38.1%)	47,430 (38.3%)	28,490 (38.9%)	24,316 (39.9%)
1	75,758 (17.8%)	7490 (17.2%)	22,369 (17.9%)	22,273 (18.0%)	13,120 (17.9%)	10,506 (17.2%)
2	57,076 (13.4%)	5563 (12.8%)	16,821 (13.4%)	17,057 (13.8%)	9920 (13.6%)	7715 (12.7%)
3	51,049 (12.0%)	4921 (11.3%)	14,808 (11.8%)	14,976 (12.1%)	8896 (12.2%)	7448 (12.2%)
4	55,459 (13.0%)	6016 (13.8%)	15,998 (12.8%)	15,764 (12.7%)	9391 (12.8%)	8290 (13.6%)
5	23,709 (5.6%)	4046 (9.3%)	7430 (5.9%)	6190 (5.0%)	3343 (4.6%)	2700 (4.4%)
Acute care						
Intravenous thrombolysis	29,556 (6.9%)	2502 (5.8%)	8840 (7.1%)	9393 (7.6%)	5118 (7.0%)	3703 (6.1%)
Endovascular thrombectomy	16,480 (3.9%)	1582 (3.6%)	5144 (4.1%)	5226 (4.2%)	2622 (3.6%)	1906 (3.1%)
Mechanical ventilation	3227 (0.8%)	1119 (2.6%)	1280 (1.0%)	518 (0.4%)	199 (0.3%)	111 (0.2%)
Tube feeding	29,162 (6.8%)	3175 (7.3%)	9763 (7.8%)	8766 (7.1%)	4205 (5.7%)	3253 (5.3%)
Fiscal year						
2014	64,972 (15.2%)	9720 (22.3%)	20,345 (16.3%)	17,233 (13.9%)	9511 (13.0%)	8163 (13.4%)
2015	70,889 (16.6%)	8016 (18.4%)	21,715 (17.4%)	19,623 (15.9%)	11,622 (15.9%)	9913 (16.3%)
2016	73,779 (17.3%)	7468 (17.2%)	21,667 (17.3%)	21,136 (17.1%)	12,545 (17.1%)	10,963 (18.0%)
2017	73,113 (17.1%)	6287 (14.5%)	21,232 (17.0%)	21,868 (17.7%)	13,051 (17.8%)	10,675 (17.5%)
2018	73,411 (17.2%)	6501 (14.9%)	20,450 (16.3%)	22,214 (18.0%)	13,324 (18.2%)	10,922 (17.9%)
2019	70,344 (16.5%)	5513 (12.7%)	19,717 (15.8%)	21,644 (17.5%)	13,119 (17.9%)	10,351 (17.0%)
Hospital volume (patients/year)						
−73	142,787 (33.5%)	19,710 (45.3%)	43,445 (34.7%)	40,364 (32.6%)	22,229 (30.4%)	17,039 (27.9%)
74–133	142,124 (33.3%)	13,599 (31.3%)	44,344 (35.4%)	43,642 (35.3%)	23,793 (32.5%)	16,746 (27.5%)
134–	141,597 (33.2%)	10,196 (23.4%)	37,337 (29.8%)	39,712 (32.1%)	27,150 (37.1%)	27,202 (44.6%)

**Table 2:** Occurrence of pneumonia according to physical rehabilitation duration.

	Intensity of Physical Rehabilitation					
	<20 min/Day (n = 43,505)	20–39 min/Day (n = 125,126)	40–59 min/Day (n = 123,718)	60–79 min/Day (n = 73,172)	≥80 min/Day (n = 60,987)	*p*-Value
Pneumonia (ICD-10: J12-J18, J69)	5849 (13.4%)	12,678 (10.1%)	10,972 (8.9%)	5384 (7.4%)	3917 (6.4%)	<0.001
Pneumonia (ICD-10: J12-J18)	2006 (4.6%)	4040 (3.2%)	3473 (2.8%)	1766 (2.4%)	1539 (2.5%)	<0.001
Aspiration pneumonia (ICD-10: J69)	3843 (8.8%)	8638 (6.9%)	7499 (6.1%)	3618 (4.9%)	2378 (3.9%)	<0.001

**Table 3:** Association between physical rehabilitation duration and pneumonia.

	Age-Sex Adjusted					Multivariate Adjusted *				
	OR	95% CI		*p*-Value	*p*-Value for Trend	OR	95% CI		*p*-Value	*p*-Value for Trend
Duration of physical rehabilitation										
<20 min/day	Reference					Reference				
20–39 min/day	0.69	0.67	0.72	<0.001	<0.001	0.78	0.75	0.81	<0.001	<0.001
40–59 min/day	0.56	0.54	0.58	<0.001		0.68	0.66	0.71	<0.001	
60–79 min/day	0.42	0.40	0.44	<0.001		0.56	0.53	0.58	<0.001	
≥80 min/day	0.33	0.31	0.35	<0.001		0.46	0.44	0.48	<0.001	

* Adjusted for age, sex, subtype of ischemic stroke, Charlson comorbidity index score, Japan Coma Scale score at admission, modified Rankin Scale score before admission, acute care type, fiscal year, and hospital case volume. OR, odds ratio; 95% CI, 95% confidence interval.

**Table 4:** Association between the duration of physical rehabilitation and non-aspiration and aspiration pneumonia.

	Age-Sex Adjusted					Multivariate Adjusted *				
	OR	95% CI		*p*-Value	*p*-Value for Trend	OR	95% CI		*p*-Value	*p*-Value for Trend
Non-aspiration pneumonia (n = 12,824)										
Duration of physical rehabilitation										
<20 min/day	Reference					Reference				
20–39 min/day	0.70	0.66	0.74	<0.001	<0.001	0.79	0.74	0.83	<0.001	<0.001
40–59 min/day	0.56	0.53	0.59	<0.001		0.68	0.64	0.72	<0.001	
60–79 min/day	0.41	0.38	0.44	<0.001		0.54	0.50	0.58	<0.001	
≥80 min/day	0.35	0.32	0.38	<0.001		0.47	0.43	0.51	<0.001	
Aspiration pneumonia (n = 25,976)										
Duration of physical rehabilitation										
<20 min/day	Reference					Reference				
20–39 min/day	0.71	0.68	0.74	<0.001	<0.001	0.80	0.77	0.84	<0.001	<0.001
40–59 min/day	0.59	0.57	0.62	<0.001		0.72	0.68	0.75	<0.001	
60–79 min/day	0.46	0.43	0.48	<0.001		0.60	0.57	0.64	<0.001	
≥80 min/day	0.35	0.33	0.37	<0.001		0.49	0.46	0.52	<0.001	

* Adjusted for age, sex, subtype of ischemic stroke, Charlson comorbidity index score, Japan Coma Scale score at admission, modified Rankin Scale score before admission, acute care type, fiscal year, and hospital case volume. OR, odds ration; 95% CI, 95% confidence interval.

## Data Availability

The datasets used and/or the analyses conducted in the current study are not publicly available for ethical reasons. The datasets are available from the corresponding author upon reasonable request, with ethical approval.

## Supplementary Materials

The following supporting information can be downloaded at: https://www.mdpi.com/article/10.3390/neurolint15040094/s1, Table S1: Association between the intensity of physical rehabilitation within 3 and 5 days of admission and non-aspiration and aspiration pneumonia; Table S2: Association between physical rehabilitation intensity and pneumonia stratified by Japan Coma Scale scores; Table S3: The association between the intensity of physical rehabilitation and pneumonia, excluding patients on mechanical ventilators within 2 days of admission (n = 423,281).
