# Supplementary material for: Physical Rehabilitation and Post-Stroke Pneumonia: A Retrospective Observational Study Using the Japanese Diagnosis Procedure Combination Database
Source: Neurol Int. 2023 Dec 4;15(4):1459–68. doi: 10.3390/neurolint15040094 (PMC10745980; doi:10.3390/neurolint15040094)
Supplement: Supplementary file 1 [file neurolint-15-00094-s001.zip › tableS1.docx]

**Table S1.** Association between the intensity of physical rehabilitation within 3 and 5 days of admission and non-aspiration and aspiration pneumonia

|  | Age-sex adjusted | | | | |  | Multivariate adjusted* | | | | |
| --- | --- | --- | --- | --- | --- | --- | --- | --- | --- | --- | --- |
|  | OR | 95% CI | | p-value | p-value for trend |  | OR | 95% CI | | p-value | p-value for trend |
| Intensity of physical rehabilitation within 3 days of admission | | | | | |  |  |  |  |  |  |
| <20 min/day | Reference | | | |  |  | Reference | | |  |  |
| 20-39 min/day | 0.78 | 0.76 | 0.81 | <0.001 | <0.001 |  | 0.84 | 0.81 | 0.86 | <0.001 | <0.001 |
| 40-59 min/day | 0.69 | 0.67 | 0.72 | <0.001 |  |  | 0.78 | 0.76 | 0.81 | <0.001 |  |
| 60-79 min/day | 0.53 | 0.51 | 0.56 | <0.001 |  |  | 0.66 | 0.63 | 0.69 | <0.001 |  |
| ≤80 min/day | 0.47 | 0.45 | 0.50 | <0.001 |  |  | 0.63 | 0.60 | 0.67 | <0.001 |  |
|  |  |  |  |  |  |  |  |  |  |  |  |
| Intensity of physical rehabilitation within 5 days of admission | | | | | |  |  |  |  |  |  |
| <20 min/day | Reference | | | |  |  | Reference | | |  |  |
| 20-39 min/day | 0.71 | 0.69 | 0.74 | <0.001 | <0.001 |  | 0.79 | 0.77 | 0.82 | <0.001 | <0.001 |
| 40-59 min/day | 0.60 | 0.58 | 0.62 | <0.001 |  |  | 0.72 | 0.70 | 0.75 | <0.001 |  |
| 60-79 min/day | 0.46 | 0.44 | 0.48 | <0.001 |  |  | 0.60 | 0.57 | 0.63 | <0.001 |  |
| ≤80 min/day | 0.37 | 0.35 | 0.39 | <0.001 |  |  | 0.51 | 0.49 | 0.54 | <0.001 |  |
| *Adjusted for age, sex, subtype of ischemic stroke, charlson comorbidity index, Japan Coma Scale score at admission, modified Rankin Scale score before admission, acute care, fiscal year, and hospital case volume. OR, odds ratio; 95% CI, 95% confidence interval. | | | | | | | | | | | |
