# Supplementary material for: Physical Rehabilitation and Post-Stroke Pneumonia: A Retrospective Observational Study Using the Japanese Diagnosis Procedure Combination Database
Source: Neurol Int. 2023 Dec 4;15(4):1459–68. doi: 10.3390/neurolint15040094 (PMC10745980; doi:10.3390/neurolint15040094)
Supplement: Supplementary file 1 [file neurolint-15-00094-s001.zip › tableS3.docx]

**Table S3.** The association between the intensity of physical rehabilitation and pneumonia, excluding patients on mechanical ventilators within 2 days of admission (n=423,281)

|  | Age-sex adjusted | | | | |  | Multivariate adjusted* | | | | |
| --- | --- | --- | --- | --- | --- | --- | --- | --- | --- | --- | --- |
|  | OR | 95% CI | | p-value | p-value for trend |  | OR | 95% CI | | p-value | p-value for trend |
| Intensity of physical rehabilitation |  |  |  |  |  |  |  |  |  |  |  |
| <20 min/day | Reference | | | |  |  | Reference | | |  |  |
| 20-39 min/day | 0.71 | 0.68 | 0.73 | <0.001 | <0.001 |  | 0.78 | 0.75 | 0.81 | <0.001 | <0.001 |
| 40-59 min/day | 0.58 | 0.56 | 0.61 | <0.001 |  |  | 0.68 | 0.66 | 0.71 | <0.001 |  |
| 60-79 min/day | 0.44 | 0.42 | 0.46 | <0.001 |  |  | 0.56 | 0.53 | 0.58 | <0.001 |  |
| ≤80 min/day | 0.35 | 0.33 | 0.37 | <0.001 |  |  | 0.46 | 0.44 | 0.49 | <0.001 |  |
| *Adjusted for age, sex, subtype of ischemic stroke, charlson comorbidity index, Japan Coma Scale score at admission, modified Rankin Scale score before admission, acute care, fiscal year, and hospital case volume. OR, odds ration; 95% CI, 95% confidence interval. | | | | | | | | | | | |
